# Isolation of a highly virulent *Metarhizium* strain targeting the tea pest, *Ectropis obliqua*

**DOI:** 10.3389/fmicb.2023.1164511

**Published:** 2023-05-15

**Authors:** Jie Zhao, Yuxi Chen, Nemat O. Keyhani, Cong Wang, Yichen Li, Huili Pu, Jincheng Li, Sen Liu, Pengyu Lai, Mengjia Zhu, Xueyou He, Shouping Cai, Xiayu Guan, Junzhi Qiu

**Affiliations:** ^1^Key Lab of Biopesticide and Chemical Biology, Ministry of Education; State Key Laboratory of Ecological Pest Control for Fujian and Taiwan Crops, College of Life Sciences, Fujian Agriculture and Forestry University, Fuzhou, Fujian, China; ^2^Department of Microbiology and Cell Science, Institute of Food and Agricultural Sciences, University of Florida, Gainesville, FL, United States; ^3^Dulwich International High School Suzhou, Suzhou, Jiangsu, China; ^4^Fujian Academy of Forestry, Fuzhou, Fujian, China; ^5^College of Horticulture, Fujian Agriculture and Forestry University, Fuzhou, Fujian, China

**Keywords:** Ascomycota, entomopathogenic fungus, *Metarhizium*, *Ectropis obliqua*, biocontrol potential, mycoinsecticide

## Abstract

**Introduction:**

Tea is one of the most widely consumed beverages around the world. Larvae of the moth, *Ectropis obliqua Prout* (Geometridae, Lepidoptera), are one of the most destructive insect pests of tea in China. *E. obliqua* is a polyphagus insect that is of increasing concern due to the development of populations resistant to certain chemical insecticides. Microbial biological control agents offer an environmentally friendly and effective means for insect control that can be compatible with “green” and organic farming practices.

**Methods:**

To identify novel *E. obliqua* biological control agents, soil and inset cadaver samples were collected from tea growing regions in the Fujian province, China. Isolates were analyzed morphologically and via molecular characterization to identity them at the species level. Laboratory and greenhouse insect bioassays were used to determine the effectiveness of the isolates for *E. obliqua* control.

**Results:**

Eleven isolates corresponding to ten different species of *Metarhizium* were identified according to morphological and molecular analyses from soil and/or insect cadavers found on tea plants and/or in the surrounding soil sampled from eight different regions within the Fujian province, China. Four species of *Metarhizium* including *M. clavatum, M. indigoticum, M. pemphigi*, and *M. phasmatodeae* were documented for the first time in China, and the other species were identified as *M. anisopliae, M. brunneum, M. lepidiotae, M. majus, M. pinghaense*, and *M. robertsii*. Insect bioassays of the eleven isolates of *Metarhizium* revealed significant variation in the efficacy of each isolate to infect and kill *E. obliqua*. *Metarhizium pingshaense* (MaFZ-13) showed the highest virulence reaching a host target mortality rate of 93% in laboratory bioassays. The median lethal concentration (LC_50_) and median lethal time (LT_50_) values of *M. pingshaense* MaFZ-13 were 9.6 × 10^4^ conidia/mL and 4.8 days, respectively. Greenhouse experiments and a time-dose-mortality (TDM) models were used to further evaluate and confirm the fungal pathogenic potential of *M. pingshaense* MaFZ-13 against *E. obliqua* larvae.

**Discussion:**

Isolation of indigenous microbial biological control agents targeting specific pests is an effective approach for collecting resources that can be exploited for pest control with lowered obstacles to approval and commercialization. Our data show the presence of four different previously unreported *Metarhizium* species in China. Bioassays of the eleven different *Metarhizium* strains isolated revealed that each could infect and kill *E. obliqua* to different degrees with the newly isolated *M. pingshaense* MaFZ-13 strain representing a particularly highly virulent isolate potentially applicable for the control of *E. obliqua* larvae.

## 1. Introduction

*Ectropis obliqua* Prout is a Lepidopteran moth species that is one of the most common insect pests found on the leaves of tea trees in China (Zeng et al., 2018). Infestations can reduce crop quality and yields, especially along the middle and lower reaches of the Yangtze River (Yan et al., 2020). *Ectropis obliqua* is a polyphagous insect with a variety of different hosts. In addition to tea, it can also cause damage to soybean [*Glycine max* (Linn.) Merr.], sesame (*Sesamum indicum* L.), and even cultivated flowers (e.g., *Chrysanthemum morifolium* Ramat. and the common sunflower *Helianthus annuus* L.) (Yang et al., 2011; Li et al., 2019).

With respect to the often hilly tea gardens, cultivation of a single tea variety coupled with optimal humidity and light (sun) cycles often leads to high infestation levels and significant damage to leaves that are still tender, moist and closed, and hence a perfect habitat for the insect, and one that makes control more difficult. The first generation of *E. obliqua* usually takes place in early April, followed by population recurrences every 6–8 weeks (Zhang et al., 2022).

Chemical, biological, and physical control methods have all been applied for suppressing *E. obliqua* populations (Idris et al., 2020). However, overuse of chemical pesticides has led to the emergence of resistance, resurgence, and residue (3R) issues (Zeng et al., 2018). This, coupled with increasing demand for organic tea, has resulted in a pressing need for the identification and development of efficient, low residue, low (non-target) toxicity control approaches for *E. obliqua* that optimally should be compatible with “green” or organic farming practices (Soh and Veera Singham, 2021). One such approach is the identification of biological control agents that has the advantages of being (i) more environmentally friendly, (ii) less harmful to humans and other animals including beneficial insects (e.g., bees and spiders), and (iii) possess decreased chances for the development of resistance (to the control method), as compared to chemical insecticides. Hence, the application of biological control is increasing for many different crop systems, especially as part of integrated pest management (IPM) practices. Nuclear polyhedrosis virus (EoNPV) and *Bacillus thuringiensis* (Bt) are currently the two most widely used biological control agents targeting *E. obliqua* (Ma et al., 2007; Idris et al., 2020). In addition, some plant-based insect toxins have been used to control *E. obliqua*, including a tea saponin that has been shown to be able to kill *E. obliqua* larvae without causing harm to non-target organisms such as *Ebrechtella tricuspidata* Fahricius and *Evarcha albaria* L. Koch (Zeng et al., 2018; Zhao et al., 2020). However, many of these approaches are costly, and application can be difficult and time-consuming. Entomopathogenic fungi represent an alternative method for targeting insects that can be cost-effective, capable of controlling target populations in diverse habitats, are naturally occurring, and have shown little, if any, development of resistance to the fungus by insect targets as a result of application (Glare et al., 2012; Ortiz-Urquiza et al., 2015; Gao et al., 2017).

Entomopathogenic fungi invade healthy insects *via* penetration of the exoskeleton, proliferation within the host body, tissue dissolution, and ultimately host death (Ortiz-Urquiza and Keyhani, 2013; Han et al., 2020; Mahato and Paikaray, 2021). There are over 100 genera and more than 1,000 species of entomopathogenic fungi described thus far (Mongkolsamrit et al., 2020; Chen et al., 2021). *Metarhizium* is one of the most important groups and includes more than 80 species (e.g., *Metarhizium anisopliae* (Metschn.) Sorokin, *Metarhizium acridum* (Driver and Milner) J.F. Bisch., S.A. Rehner and Humber, *Metarhizium robertsii* J.F. Bisch., S.A. Rehner and Humber, *Metarhizium clavatum* Luangsa-ard, Mongkols., Lamlertthon, Thanakitp. and Samson, *Metarhizium indigoticum* (Kobayasi and Shimizu) Kepler, S.A. Rehner and Humber, and *Metarhizium pemphigi* (Driver and Milner) Kepler, Humber and S.A. Rehner). Some of these are species that are cosmopolitan and have a broad host range as well as those that have narrower host ranges. For example, *M. anisopliae* can infect > 200 different species of insects throughout the world, while *M. acridum* is mainly a pathogen of members of the Acrididae. The former is used in a wide variety of crop applications, whereas the latter has been commercialized mainly for the control of locusts (Mongkolsamrit et al., 2020; St Leger and Wang, 2020; Kamga et al., 2022).

Known entomopathogenic fungi that can target *E. obliqua* include *Paecilomyces* spp., *Fusarium* spp., and *Erynia* spp. as well as *Beauveria bassiana* (Balsamo) Vuillemin and *Metarhizium anisopliae* (Idris et al., 2020; Wang et al., 2020; Zhang et al., 2020). However, aside from *M. anisopliae*, other species of *Metarhizium* infecting *E. obliqua* have yet to be reported. Here, we report on the field isolation and screening of entomopathogenic fungi, identifying 11 *Metarhizium* isolates belonging to 10 different species. Our data provide records for species resources, identify new taxa in China, and examine the biological control potential of all of the isolates against *E. obliqua* larvae as compared with the reference *M. anisopliae* (CQMa421) strain currently commercialized for a range of target insects. These data identify one isolate, *M. pingshaense* MaFZ-13, as particularly effective in laboratory bioassays toward *E. obliqua*, and extend these results to greenhouse experiments that confirm the effectiveness of the fungal strain for *E. obliqua* control.

## 2. Materials and methods

### 2.1. Fungal acquisition

Eleven fungal isolates were gathered from moribund insect cadavers or obtained from soil samples in Fujian Province (23°33′-28°19′N, 116°15′-120°43′E), China. Collection sites encompassed a range of tea-growing areas including the Wuyi Mountain Nature Reserve of Wuyi city, Wanmulin Nature Reserve of Jian'ou city, Taimu Mountain Nature Reserve of Ningde city, Huangchu Forest Nature Reserve, Gushan Mountain and Fuzhou National Forest Park of Fuzhou city, Niumulin Nature Reserve of Quanzhou city, and Hubo Liao Nature Reserve of Zhangzhou city (Supplementary Figure S1).

### 2.2. Fungal isolation

Field-collected insect cadavers, typically fully covered with fungal mycelia, and soil samples were collected from the different natural reserves. Collected insects were cut or clipped with a knife or tweezers on a sterile worktable (Airtech SW-CJ-2FD). Samples were soaked in 75% alcohol for 30–60 s, rinsed in sterile water for 10–15 s, then placed on a clean glass slide, and slightly smashed with a clean blade or the back of the blade handle until the base was crushed, after which 200 μL of sterile distilled dH_2_O was added, mixed with the crushed materials, and then placed onto potato dextrose agar (PDA, Difco TM) Petri dishes (Bioshap) and cultured at 25 ± 1°C and 60% relative humidity (RH) for 10–14 days in an incubator (Binder BD240). Soil samples were serially diluted using sterile ddH_2_O and spread onto PDA plates. Colonies were assessed after 3–5 d growth, and single colonies were gently picked and streaked onto fresh culture media for several rounds until the resultant single colonies were homogeneous in appearance (Kumar et al., 2018; Wei et al., 2019).

### 2.3. Identification of fungi

#### 2.3.1. Morphological identification

Single colony purified strains were inoculated in the center of PDA plates and allowed to grow. Asexual characteristics, including the production and morphology of phialides, mycelia, and conidia, were examined by inoculating spores on glass slides containing a thin layer of PDA (5–10 mm in diameter) and overlaid with a cover slip (Kepler et al., 2014; Mongkolsamrit et al., 2020). Slides were placed in Petri dishes with a small amount of water to maintain humidity and kept at room temperature to visualize the development of phialides and conidia. Where indicated, 1–2 drops of cotton blue were added to the slide, and sample blocks were encapsulated with colorless transparent nail polish for further observation. Micromorphological images of the fungal cells (e.g., size and shape) were collected using a Nikon Eclipse 80i microscope (Nikon) attached to a digital camera. Twenty to thirty individual length and width measurements were taken for each sample, with absolute minima and maxima. Mycelia were inoculated on PDA plates and incubated at 25°C with colonies photographed and growth diameters measured as indicated (Kepler et al., 2014; Mongkolsamrit et al., 2020).

#### 2.3.2. Gene amplification and molecular phylogenetic analysis

The Omega Bio-Tek fungal kit was used to extract fungal total DNA, and primers were used to amplify (1) transcription elongation factor (*TEF*) and (2) the RNA polymerase II largest subunit 1 (*RPB1*) gene fragments of the fungal strains based on previous reports (Kepler et al., 2014; Senthil Kumar et al., 2021). Primers 983F (5′-GCYCCYGGHCAYCGTGAY TTYAT-3′) and 2218R (5′-ATGACACCRACRGCRACRGTYTG-3′) were used for the *TEF* gene, and primers *RPB1* (cRPB1Af 5′-CAYCCWGGYTTYATCAAGAA-3′ and RPB1Cr 5′-CCNGCDATNTCRTTRTCCATRTA-3′) were used for *RBP1* amplification (Senthil Kumar et al., 2021). In brief, polymerase chain reaction (PCR) experiments were performed as follows: (1) for *TEF* amplification: 95°C for 10 min, followed by 40 cycles at 94°C for 30 s, 55°C for 30 s, 72°C for 1 min, and a final extension step at 72°C for 10 min; (2) for *RPB1* amplification: 95°C for 5 min, followed by 40 cycles at 95°C for 1 min, 50°C for 2 min, 72°C for 2 min, and a final extension step at 72°C for 10 min. The resultant PCR products were sequenced at Boshan Company, China. The generated sequences were submitted to NCBI GenBank and used for multi-gene phylogenetic analysis. The sequences were employed as the query to perform a BLAST search (http://blast.ncbi.nlm.nih.gov/), and the sequence similarity studies were performed using ClustalX. Phylogenetic trees were constructed with the method of Bayesian inference (BI) using MrBayes software (Portela et al., 2021).

### 2.4. Insect breeding

A laboratory colony of *E. obliqua* was established using late instar larvae gathered from an infested tea plantation in Fuzhou National Forest Park, Fuzhou, China. Insects were fed on the fresh tender tea leaves and maintained in plastic rearing cages (30 × 30 × 30 cm) and kept at 25 ± 2°C, 70–80% relative humidity (RH) with 10:14 h (day: night) photoperiod until pupation. Pupae were gently picked from leaves and transferred into a spawning container (15 × 15 × 20 cm) and raised with 10% honey water until eclosed adults were seen. After adult emergence and mating, the adults were allowed to lay eggs on fresh young tea leaves grown inside the cages. Larvae were reared on the new tender leaves after hatching and collected for use in bioassays at the second instar stage **(**Lu et al., 2020).

#### 2.5. Virulence bioassays

##### 2.5.1. Conidial preparation

All fungal strains were inoculated on PDA medium and incubated in an incubator at 25 ± 1°C and 60% RH for 14 days. Spores produced on PDA Petri dishes were harvested into sterile distilled water containing 0.05% Tween 80 (Sigma-Aldrich), and the conidial solution was mixed until uniform. The spore suspension was then filtered through 4–8 layers of lens paper and spore concentrations were determined using a hemocytometer. Spore concentrations were then adjusted as indicated. For the determination of the fungal median lethal concentration to kill 50% of insect hosts, (LC_50_), five concentrations of fungal spores: 1 × 10^4^, 1 × 10^5^, 1 × 10^6^, 1 × 10^7^, and 1 × 10^8^ conidia/mL, were used. For calculation of the median lethal time to kill 50% of the insect hosts, (LT_50_), a suspension of 1 × 10^8^ conidia/mL was used.

##### 2.5.2. Insect bioassays

*Ectropis obliqua* 2nd instar larvae were placed on fresh tea leaves, and spore suspension adjusted to different concentrations as above was evenly sprayed onto the surface of the test insect, which was then stored and maintained in a sterilized feeding cage (AIPUINS) after allowing the insects to air dry after treatment. Each treatment consisted of 20 larvae × three technical replicates for each group. Sterile distilled water containing 0.05% Tween 80 only was served as a control. The feeding cages were continuously cultured in a greenhouse at 25 ± 2°C, relative humidity (70–80%), and 10:14h (day: night) for lab assay. The number of dead larvae (surface melanization of insect cadaver, followed by sporulation) was counted daily after 24 h of post-inoculation, and the test was terminated 13 days after treatment. The dead larvae were picked out and placed in a clean plastic petri dish without media to observe fungal sporulation. The mortality rate of the control group was <10% (Im et al., 2022). The entire experiment was repeated three times with different batches of insects and spores.

##### 2.5.3. Greenhouse experiments

Three greenhouse trials with *M. pingshaense* were conducted from March to November 2022, respectively, in *E. obliqua*-infested tea seedlings planted in the greenhouse (temperature 25 ± 2°C, 70–80% RH, 10:14 h day: night). The 2nd instar larval numbers of *E. obliqua* per seedling were counted with a counter before fungal spray (Beloti et al., 2015). The fungal spore suspensions (1 × 10^4^, 1 × 10^5^, 1 × 10^6^, 1 × 10^7^, and 1 × 10^8^ conidia/mL) were prepared in sterile distilled water containing 0.05% Tween 80 (Sigma-Aldrich). The spore suspensions of 500 mL at the above different concentrations were used to thoroughly spray each tea tree (*n* = 20) that was naturally infested with larvae of *E. obliqua* with the aid of 2-liter pump action handheld sprayer (Senthil Kumar et al., 2021). Twenty tea seedlings sprayed with sterilized water containing 0.05% Tween 80 acted as a control (Beloti et al., 2015). Mortality data were counted daily according to the treated and control plants and terminated after 10 days of treatment (Senthil Kumar et al., 2021). The greenhouse trials were repeated three times, and greenhouse bioassays were performed in triplicate at different time intervals. The mortality rates were calculated using the recorded data from each treatment as mentioned above.

#### 2.6. Statistical analysis

Data were transformed by arc sine [(x + 0.5)/100] ½ before analysis (Senthil Kumar et al., 2021). Data on conidial concentration, time, and their interactive effects on insect mortality rate were analyzed using GLM. Mortality data of *Ectropis oblique* were corrected using Abbott's formula. Median lethal concentration (LC_50_) and median lethal time (LT_50_) values were calculated by DPS software based on the binomial GLM function. Biocontrol efficacy data were estimated with the aid of the Student's *t*-test and TDM model (Tang and Zhang, 2013; Lian et al., 2021).

## 3. Results

### 3.1. Species identification of *Metarhizium* spp.

#### 3.1.1. Morphological identification

A total of 11 *Metarhizium* isolates, collected from fungal-infected cadavers of insects found on tea [*Camellia sinensis* (Linnaeus) Kuntze] leaves and from soil samples from various tea-growing regions in the Fujian Province of China, were single colony purified, and their morphology and the molecular sequences for the *TEF* and *RBP1* genes were determined as detailed in the Methods section (Kepler et al., 2014; Mongkolsamrit et al., 2020). A summary of the host/origin, GenBank accession numbers for the cloned *TEF* and *RBP1* regions, and species assignments for the 11 *Metarhizium* isolates are given in Supplementary Table S1. Isolates were plated onto PDA, and sporulation for all isolates was observed beginning ~4–6 days after initial inoculation. Morphological characteristics (Supplementary Table S2) were recorded as follows:

##### 3.1.1.1. *Metarhizium anisopliae*

**Figure 1:**
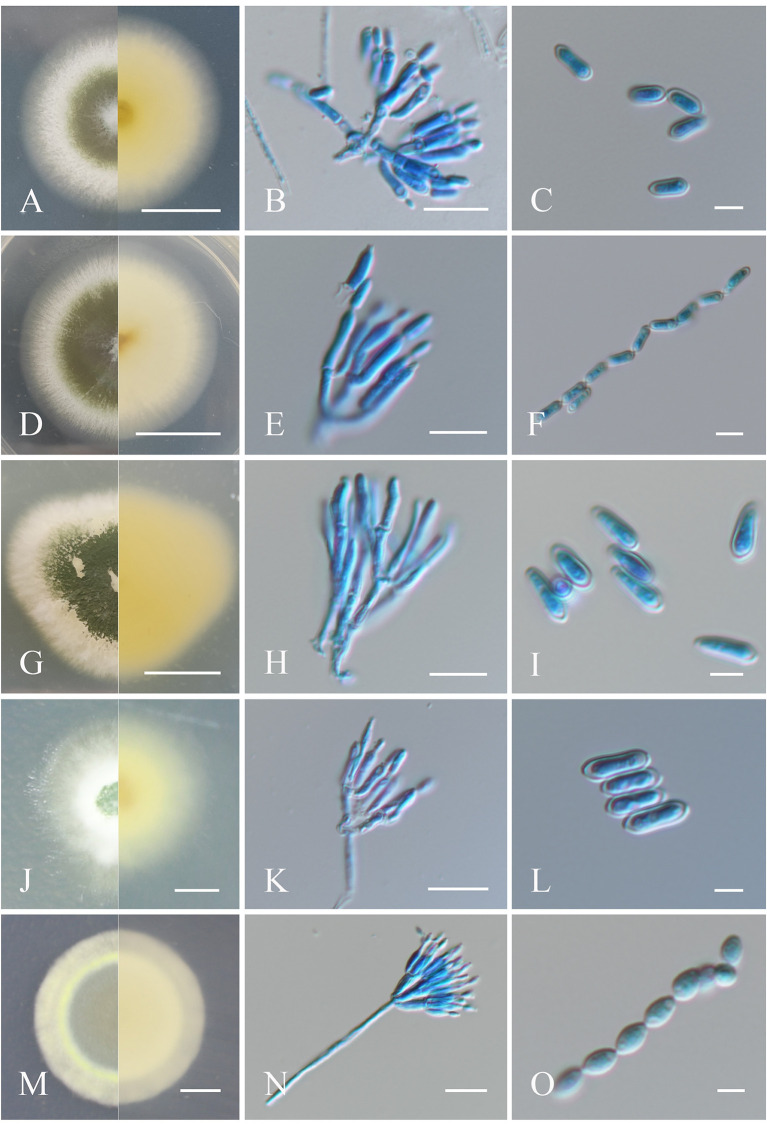
Morphological characteristics of the isolates of *Metarhizium*. **(A, D, G, J, M)** Colonies of *M. anisopliae, M. pingshaense, M. robertsii, M. majus*, and *M*. *brunneum* cultured on PDA at 25°C for 14 days; **(B, E, H, K, N)** spore production structures of each isolate stained with cotton blue, respectively; **(C, F, I, L, O)** conidial morphology of each isolate stained with cotton blue, respectively. Scale bars: **(A, D, G, J, M)** = 0.5 cm; **(B, E, H, K, N)** = 10 μm; **(C, F, I, L, O)** = 5 μm.

Fungal colonies grown on PDA at 25°C for 14 days, which had a diameter of 50 mm, initially seemed white and gradually turned greenish olive, with the reverse side dark yellow to white. Sporulation for all isolates was observed beginning ~4–6 days after initial inoculation. Conidiophores were born in aerial mycelia and were smooth, terete to clave-like, apically branched, 2–3 phialides on per branch, (5–) 7.0–9.0 (−10.5) × 2–2.5 (−3) μm (Kepler et al., 2014). Conidia were cylindrical or clavate, smooth-walled, adhering in globose to cylindrical head at the apex of phialides, (5–) 6.0–8.0 (−9) × 2–2.5 (−3) μm (Figures 1A–C).

##### 3.1.1.2. *Metarhizium pingshaense*

Colonies of the fungus cultured on PDA, which had a diameter of 48 mm at 25°C for 14 days, initially appeared white and gradually changed dark green to olive after 4–6 days of sporulation, with the reverse light yellow to white (Mongkolsamrit et al., 2020). Conidial production was concentrated at the center of the colony and formed a concentric circular pattern toward the end. Conidiophores were born in aerial mycelia and were smooth, terete, apically branched, 1–3 phialides on per bifurcation, (5–) 6.5–8.5 (−10) × 2–3 μm. Conidia were cylindrical or clavate in shape, formed in long chains, and appeared smooth-walled, (5–) 6.0–7.0 (−8.5) × 1.5–2 (−2.5) μm (Figures 1D–F).

##### 3.1.1.3. *Metarhizium robertsii*

Fungal colonies formed on PDA, with a diameter of 31 mm at 25°C for 14 days, changed from flocky and white cream to green over time, with pale yellow on the reverse side after sporulation (Mongkolsamrit et al., 2020). Conidiophores emerged on aerial hyphae and were branched, with smooth walls. Phialides were cylindrical and slightly curved at the top, (5–) 6.5–8.5 (−11) × 1.5–2 μm. Conidia were clavate, smooth-walled, adhering in globose to cylindrical heads at the apex of phialides, (5.0–) 7.0–8.0 (−9.0) × 2–2.5 (−3) μm (Figures 1G–I).

##### 3.1.1.4. *Metarhizium majus*

Fungal colonies incubated on PDA at 25°C for 14 days were ~29 mm in diameter and were initially white and hairy, progressively developing into dark green due to conidia production, with light yellow to white on the reverse (Mongkolsamrit et al., 2020). Conidiophores were apically branched, with 2–3 phialides. Phialides were cylindrical in shape with semi-mastoid tips, (6.0–) 7–9 (−10) × 2–3 μm. Conidia were cylindrical and smooth-walled, (5–) 6.5–8.0 (−10) × 2.5–3 μm (Figures 1J–L).

##### 3.1.1.5. *Metarhizium brunneum*

Colonies on PDA at 25°C for 14 days were ~30 mm in diameter, originally appearing white and turning grayish-green upon sporulation, with white to pale yellow coloration on the reverse. Conidiophores were generated on aerial mycelium and were smooth, apically branched, and cylindrical, each with 2–3 phialides. Phialides walls were smooth, cylindrical to fusiform in shape, and slightly raised at the top, (4.5–) 5–6 (−7) × 1.5–2 μm. Conidia were smooth-walled, oval in shape, and formed in long chains, 3.5–4 (−5) × 2–2.5 (−3) μm (Mongkolsamrit et al., 2020; Figures 1M–O).

##### 3.1.1.6. *Metarhizium clavatum*

**Figure 2:**
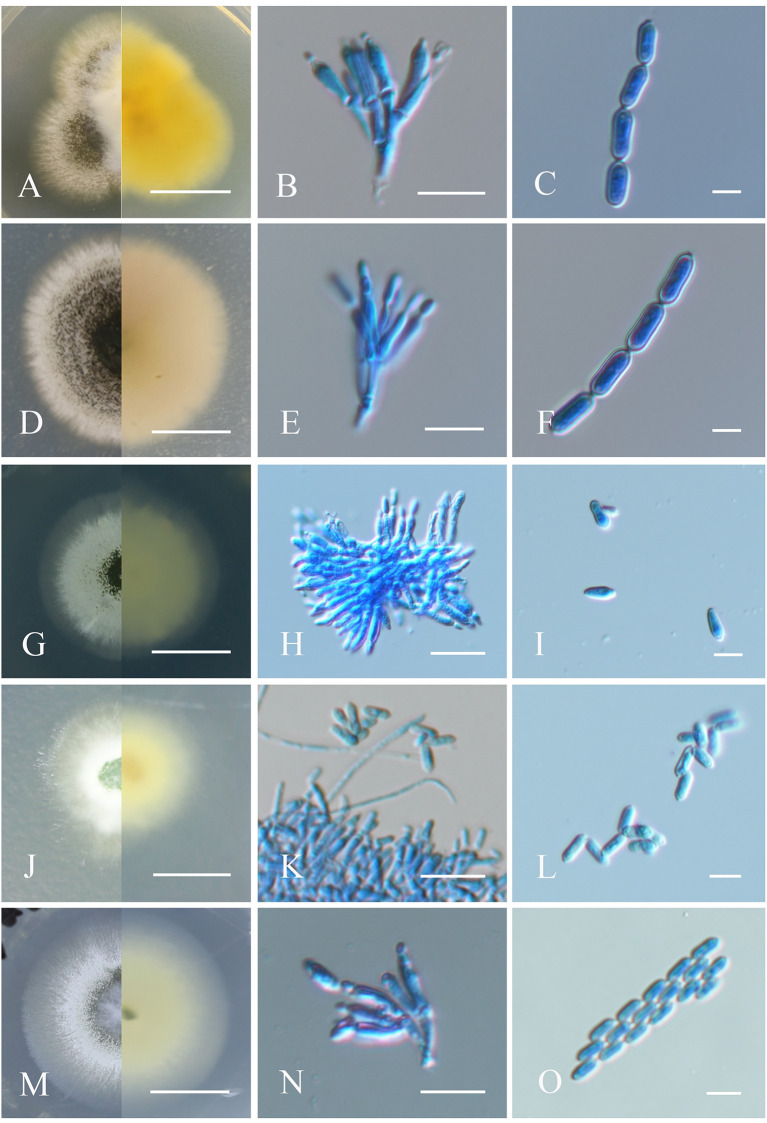
Morphological characteristics of the isolates of *Metarhizium*. **(A, D, G, J, M)** Colonies of *M. clavatum, M. indigoticum, M. phasmatodeae, M. pemphigi*, and *M. lepidiotae* cultured on PDA at 25°C for 14 days; **(B, E, H, K, N)** spore production structures of each isolate stained with cotton blue, respectively; **(C, F, I, L, O)** conidial morphology of each isolate stained with cotton blue, respectively. Scale bars: **(A, D, G, J, M)** = 0.5 cm; **(B, E, H, K, N)** = 10 μm; **(C, F, I, L, O)** = 5 μm.

Fungal colonies on PDA medium at 25°C for 14 days were ~17 mm in diameter and gradually changed from white to yellowish green upon sporulation, with the reverse side orange-yellow. Conidiophores were generated on branches and formed palisade layers. Phialides were cylindrical with a semi-mastoid tip, (4–) 6–8 (−9) × (1.5–) 2–3 μm (Mongkolsamrit et al., 2020). Conidia were cylindrical, smooth-walled, and formed in long chains, adhering in globose to cylindrical heads at the apex of phialides, (4–) 5–6 (−8) × 2.5–3 μm (Figures 2A–C).

##### 3.1.1.7. *Metarhizium indigoticum*

Fungus colonies on PDA at 25°C originally appeared white, villous, and aerial and gradually became dark brown and velvety, with radial cracks and nearly colorless reverse after 4–6 days of sporulation, with a colony diameter of ~30 mm after 14 days. Conidiophores are apically branched, each with 2–3 phialides (Kepler et al., 2014). Phialides were cylindrical or flask-shaped with a half mastoid tip, 6–8 (−9) × 2–2.5 (−3) μm. Conidia were oval, smooth-walled, and formed in long chains, 6.5–8 (−9.5) × 2–3 μm (Figures 2D–F).

##### 3.1.1.8. *Metarhizium phasmatodeae*

Colonies cultivated on PDA medium initially appeared white, smooth, and cottony and progressively turned leaf green to olive upon sporulation with pale yellow reverse coloring, and a colony diameter of ~40 mm after 14 days. Conidiophores were produced on aerial mycelium and were smooth, cylindrical, and branched (Mongkolsamrit et al., 2020). Phialides were smooth-walled, cylindrical to clubbed in shape, and slightly opacified at the top, (5–) 7.0–9.0 (−10) × 2–3 μm. Conidia were cylindrical or clavate, smooth-walled, adhering in globose to cylindrical head at the apex of phialides, (5–) 6.5–8.0 (−9) × 2–2.5(−3) μm (Figures 2G–I).

##### 3.1.1.9. *Metarhizium pemphigi*

Colonies on PDA originally appeared white and cottony and gradually turned light green and leaf green with pale yellow reverse side after producing spores, and a colony diameter of ~20 mm after 14 days (Kepler et al., 2014). Conidiophores formed on aerial mycelium and were smooth and cylindrical in shape, 5–6 (−7) × 1.5–2 μm. Conidia were cylindrical or clavate, smooth-walled, adhering in globose to cylindrical heads at the apex of phialides, 4–5 (−6) × 1.5–2 μm (Figures 2J–L).

##### 3.1.1.10. *Metarhizium lepidiotae*

Colonies on PDA were initially white, flocculent, and cotton-like and gradually turned dark brown after sporulation with pale yellow coloring on the reverse, and a colony diameter of ~25 mm after 14 days. Conidial generation was denser in the middle of the colony and formed a concentric circular pattern toward the end. Conidiophores occurred on aerial hyphae and were smooth-walled, with apical branches, and 2–3 phialides per branch. Phialides were smooth-walled, clave-like with semi-mastoid tips, (6–) 6.5–9.5 (−10) × 2–3 μm. Conidia were smooth-walled, cylindrical, and grew in long parallel chains, 5–6 (−7) × 2–2.5 μm (Mongkolsamrit et al., 2020; Figures 2M–O).

#### 3.1.2. Molecular phylogeny

**Figure 3:**
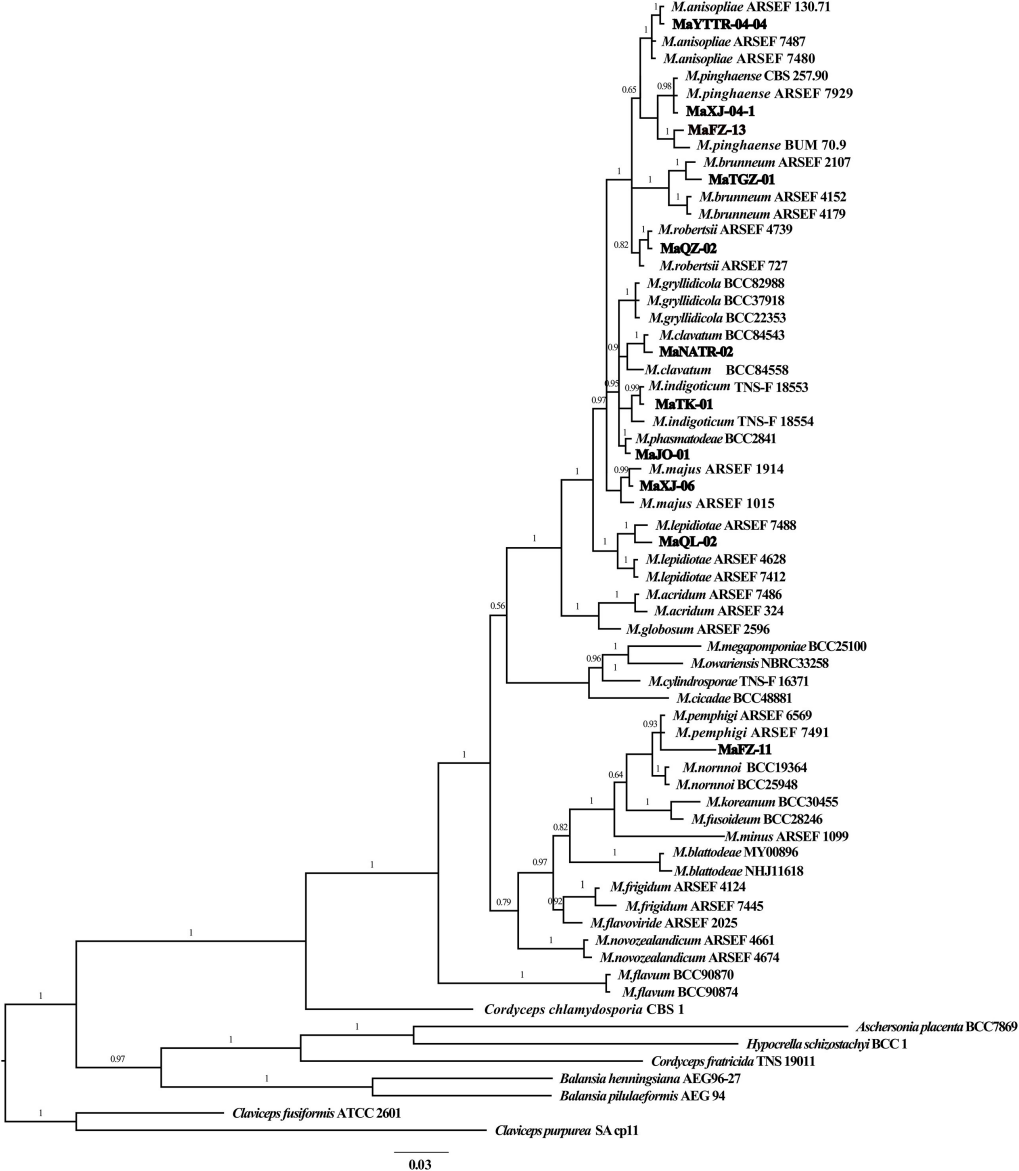
Phylogenetic tree of *Metarhizium* constructed using Bayesian inference analyses of combined *TEF* and *RPB1* sequence data.

The partial nucleotide sequences of the *TEF* and *RBP1* conserved regions of the fungal isolates were amplified in the present study to validate their morphological identities and deposited at GenBank with accession numbers (Supplementary Table S1). BLAST analyses of generated sequences exhibited high sequence similarity with different species of *Metarhizium*. A phylogenetic tree of *Metarhizium* at the species level was constructed based on the Bayesian inference (BI) method using a combined data set of RPB1 and TEF, with a combined gene length of 1,102 bp. *Metarhizium anisopliae* MaYTTR-04-04, MaXJ-04-1 and MaFZ-13 of *M. pinghaense, M. brunneum* MaTGZ-01, *M. robertsii* MaQZ-02, *M. clavatum* MaNATR-02, *M. indigoticum* MaTK-01, *M. majus* MaXJ-06, *M. lepidiotae* MaQL-02, *M. pemphigi* MaFZ-11, and *M. phasmatodeae* MaJO-01 were grouped in their individual species clades, with the 11 test isolates clearly identified within the 10 established species (Figure 3). Notably, *M. clavatum, M. indigoticum, M. pemphigi*, and *M. phasmatodeae* were newly discovered and recorded in China for the first time. Overall, the 11 *Metarhizium* isolates were placed into 10 different species, with two exemplars of *M. pingshaense*.

### 3.2. Bioassay

**Figure 4:**
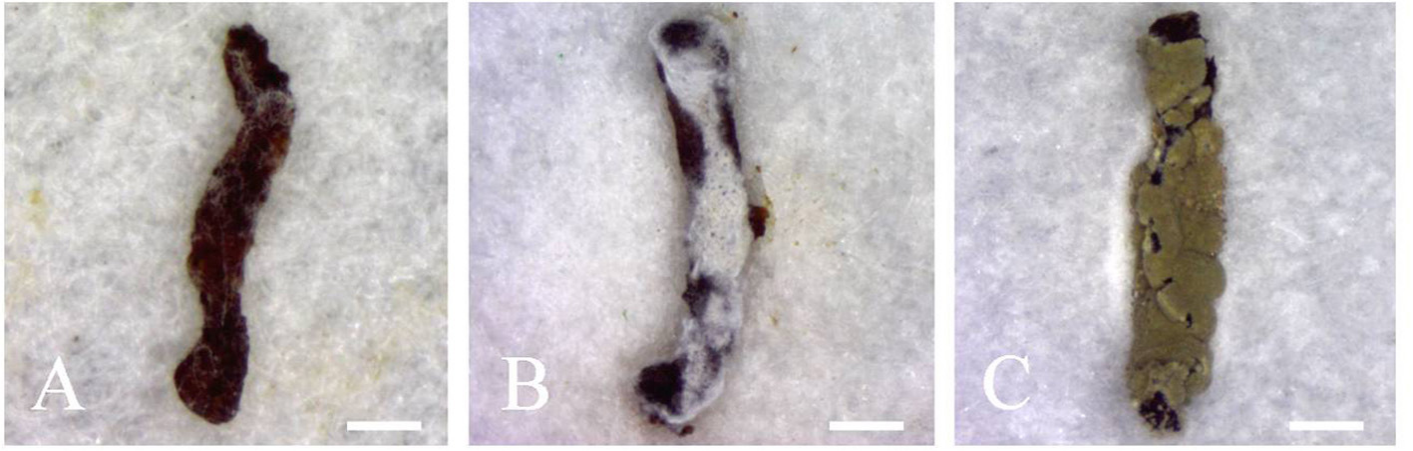
Infected second instar larvae of *E. obliqua* showing **(A)** melanization (1–2 d post-inoculation), **(B)** proliferation of hyphae on the surface of the insect (2–4 d post-inoculation), and **(C)** massive sporulation on the insect corpses. Scale bars: **(A–C)** = 1 mm.

Preliminary fungal screening using *E. obliqua* 2nd instar larvae and fungal spore concentrations of 5 × 10^9^ conidia/mL led to 100% pest mortality 10 days post-inoculation for three randomly selected *Metarhizium* isolates tested. Infected insects initially showed melanization (1–2 d post-inoculation, Figure 4A), followed by a gradual covering of the body surface with white hyphae (2–4 d post-inoculation, Figure 4B), ultimately resulting in the whole body of the insect cloaked with thick layers of green conidia (3–5 days after insect death, Figure 4C).

**Figure 5:**
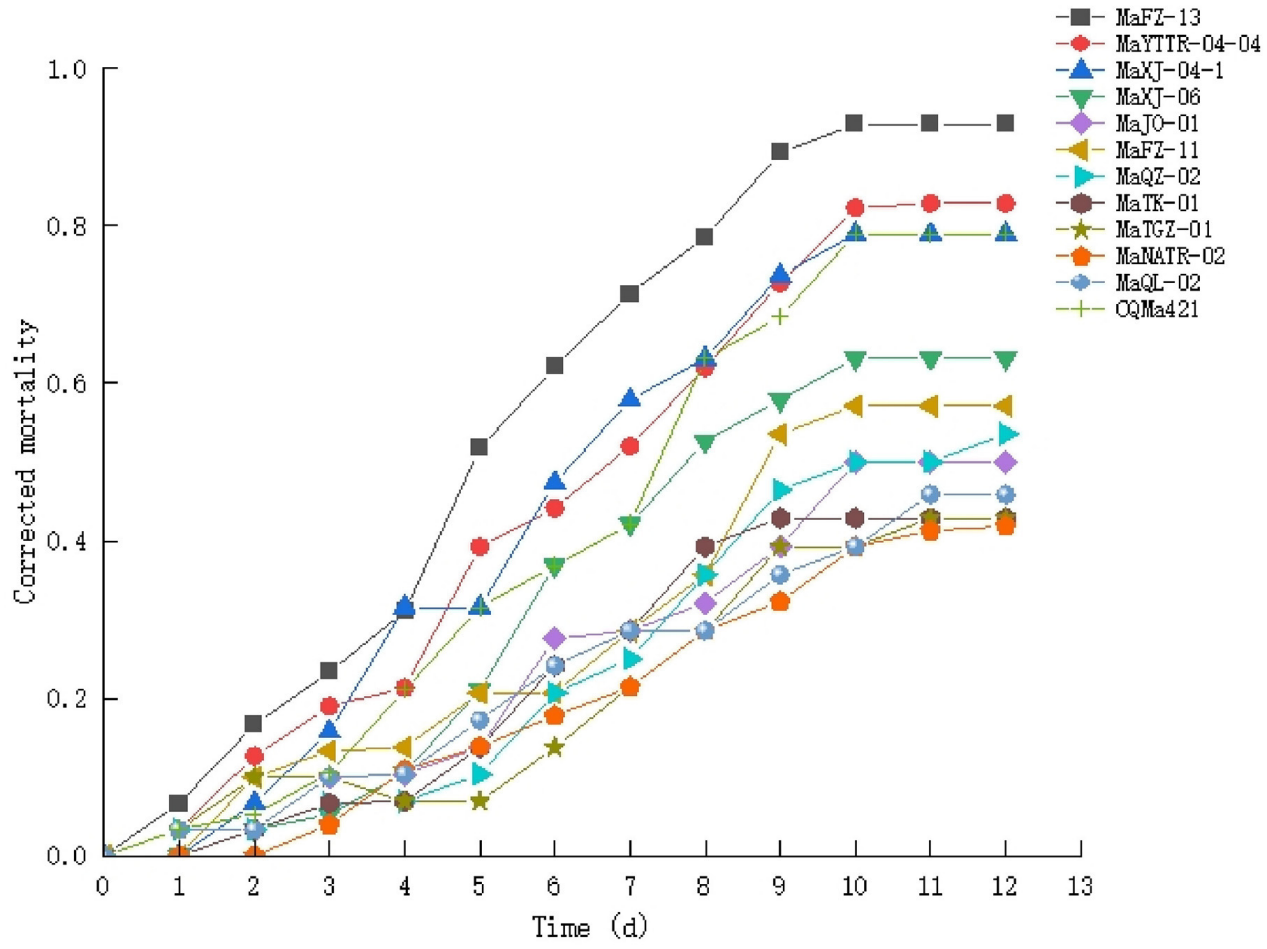
Time course of insect bioassays using the isolates of *Metarhizium* (1 × 10^8^ conidia/ml) and *E. obliqua* 2nd instar larvae as the host. Experiments were performed in triplicate. Error bars = ±SE.

**Figure 6:**
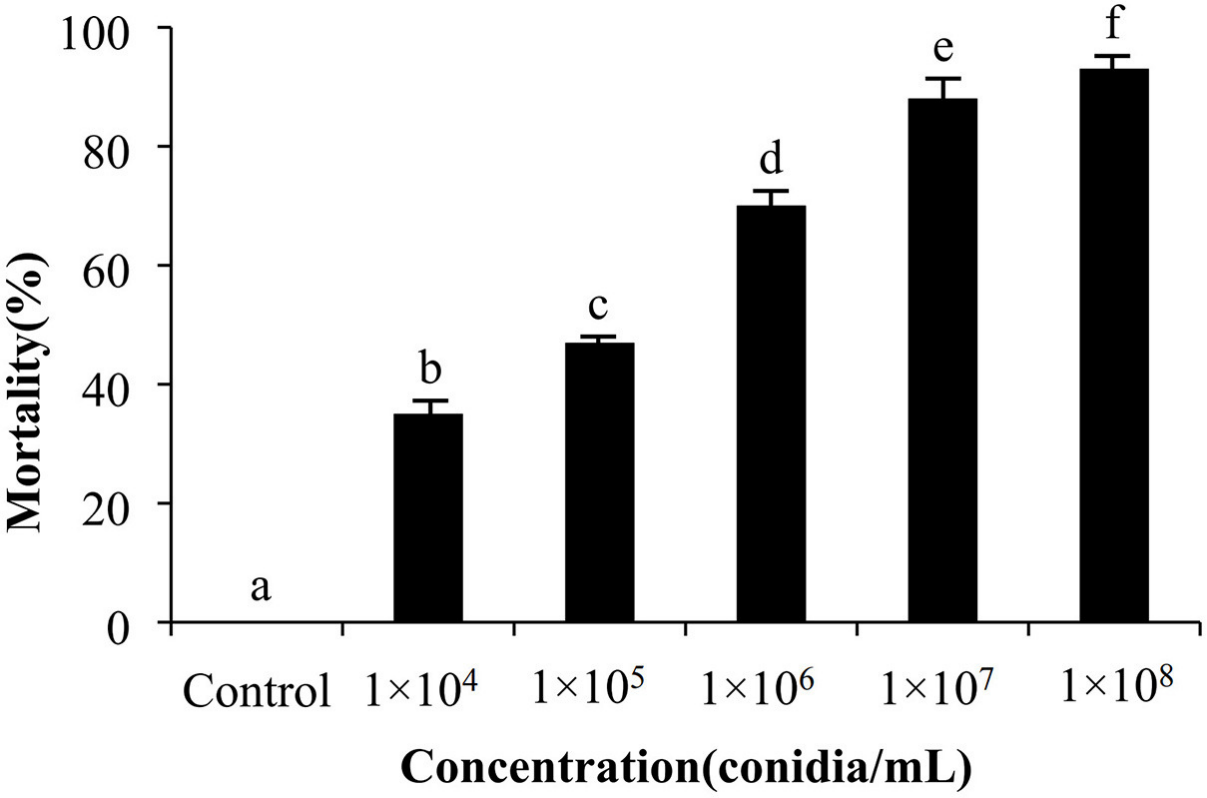
Concentration course of insect bioassays using the *M. pingshaense* (1 × 10^4^ to 1 × 10^8^ conidia/ml) and *E. obliqua* 2nd instar larvae as the host. Experiments were performed in triplicate. Error bars = ± SE. Mortalities followed by different letters of alphabets are significantly different at *P* < 0.05.

A second round of bioassays using *E. obliqua* 2nd instar larvae and fungal spore concentrations of 1 × 10^8^ conidia/mL was carried out over the course of 13 days (Figure 5). Among these, infection using *M. pingshaense* MaFZ-13 resulted in the highest mortality toward *E. obliqua* larvae reaching 93%, followed by that of *M. anisopliae* MaYTTR-04-04 (83% mortality) and *M. pingshaense* strain MaXJ-04-1 (79% mortality). *Metarhizium clavatum* MaNATR-02 showed the lowest virulence to *E. obliqua* with approximately 40% mortality after 13 days post-inoculation, with several others showing similar levels of mortality ranging from 40 to 60% of the target insects killed within the bioassay time frame, and those of the other species of *Metarhizium* fell between the two. The calculation of LT_50_ values confirmed the highest virulence for *M. pingshaense* MaFZ-13, LT_50_ = 4.8 ± 0.1 d, and lowest for *M. indigoticum* MaTK-01, LT_50_ = 9.9 ± 0.3 d (Table 1). Additional bioassays were performed in order to calculate the mean lethal concentration to kill 50% (LC_50_) of the target insect 10 days post-inoculation using different conidial concentrations ranging from 1 × 10^4^ to 1 × 10^8^ conidia/mL (Figure 6; Table 2). The LC_50_ for *M. pingshaense* MaFZ-13 was calculated to be LC_50_ = 9.6 ± 0.4 × 10^4^ conidia/mL, with LC_50_ values for other strains = 1.3 ± 0.2 × 10^8^ conidia/mL (*M. brunneum* MaTGZ-01, lowest virulent strain), and ranging from 5.2 ± 0.2 × 10^5^ to 4.8 ± 0.3 × 10^7^ conidia/mL for the remaining strains. For comparative purposes, the commercially available *M. anisopliae* strain CQMa421 was used in bioassays against *E. obliqua* 2nd instar larvae, showing an LT_50_ = 5.2 ± 0.2 d and an LC_50_ = 8.5 ± 0.6 × 10^5^ conidia/mL (Tables 1, 2). The highest mortality was seen for the isolate corresponding to *M. pingshaense*, suggesting that it may hold promise for *E. obliqua* control with greater efficiency than current strains.

**Table 1:** Calculated mean lethal time (LT_50_) values of different strains of *Metarhizium* against 2nd instar *E. obliqua* larvae using 1 × 10^8^ conidia/mL.

**Strains**	**X^2^**	**LT_50_**	**LT**_**50**_ **confidence interval**	
			**Lower**	**Upper**
MaTGZ-01	1.0183	9.879	7.5385	17.5088
MaJO-01	0.7253	8.03	6.511	11.7208
MaQZ-02	2.8616	8.3733	6.5363	13.3117
MaTK-01	2.3667	9.9117	7.319	18.9122
MaXJ-06	0.8745	6.1136	5.053	7.9948
MaNATR-02	0.4618	8.7713	6.6215	15.3126
MaYTTR-04-04	3.0707	5.0074	4.1845	6.1239
MaXJ-04-1	1.7733	5.0831	4.2288	6.2713
MaFZ-11	1.8264	8.6215	6.6596	14.1465
MaQL-02	1.4178	9.4151	7.0408	17.137
MaFZ-13	3.5715	4.8095	4.2803	5.4512
CQMa421	1.9641	5.1628	4.2053	6.2357

**Table 2:** Calculated mean lethal concentration (LC_50_ and LC_90_) values of different strains of *Metarhizium* against 2nd instar *E. obliqua* larvae.

**Strains**	**X^2^**	**LC_50_**	**LC**_**50**_ **confidence interval**		**LC_90_**	**LC**_**90**_ **confidence interval**	
			**Lower**	**Upper**		**Lower**	**Upper**
MaTGZ-01	0.1899	1.34 × 10^8^	6.38 × 10^6^	3.87 × 10^15^	2.15 × 10^16^	5.37 × 10^10^	3.77 × 10^107^
MaJO-01	0.0366	9.61 × 10^6^	1.19 × 10^6^	5.53 × 10^9^	5.59 × 10^13^	6.52 × 10^9^	1.77 × 10^48^
MaQZ-02	0.3044	8.75 × 10^6^	1.18 × 10^6^	2.51 × 10^9^	1.97 × 10^13^	4.72 × 10^9^	3.23 × 10^40^
MaTK-01	0.2126	4.78 × 10^7^	3.60 × 10^6^	3.62 × 10^12^	1.31 × 10^15^	2.21 × 10^10^	8.85 × 10^69^
MaXJ-06	0.3083	3.78 × 10^6^	6.96 × 10^5^	1.20 × 10^8^	5.81 × 10^11^	1.24 × 10^9^	1.67 × 10^25^
MaNATR-02	0.0519	2.76 × 10^7^	2.82 × 10^6^	7.81 × 10^10^	8.33 × 10^13^	9.31 × 10^9^	2.91 × 10^45^
MaYTTR-04-04	1.5224	5.20 × 10^5^	1.42 × 10^5^	1.97 × 10^6^	6.86 × 10^8^	4.57 × 10^7^	9.23 × 10^11^
MaXJ-04-1	0.9677	1.08 × 10^6^	3.15 × 10^5^	5.20 × 10^6^	1.87 × 10^9^	8.86 × 10^7^	9.08 × 10^12^
MaFZ-11	0.3045	3.11 × 10^6^	5.87 × 10^5^	7.81 × 10^7^	4.10 × 10^11^	1.04 × 10^9^	2.53 × 10^24^
MaQL-02	0.3031	4.59 × 10^7^	3.16 × 10^6^	1.83 × 10^13^	6.00 × 10^15^	3.20 × 10^10^	1.01 × 10^96^
MaFZ-13	1.4632	9.57 × 10^4^	1.94 × 10^4^	2.92 × 10^5^	3.00 × 10^7^	5.34 × 10^6^	1.24 × 10^9^
CQMa421	1.1368	8.52 × 10^5^	2.71 × 10^5^	4.35 × 10^6^	1.28 × 10^9^	7.43 × 10^7^	5.69 × 10^12^

### 3.3. Greenhouse trial and TDM evaluation of MaFZ-13

The cumulative mortality of *E. obliqua* larvae was recorded over a 10-day period of treatment with different concentrations (1 × 10^4^ to 1 × 10^8^ conidia/mL) of *M*. *pingshaense* MaFZ-13 in a greenhouse setting as detailed in the Methods section. The population of *E. obliqua* larvae decreased by 93, 78, 65 43%, and 28% 10 days post-inoculation using 1 × 10^8^, 1 × 10^7^, 1 × 10^6^, 1 × 10^5^, and 1 × 10^4^ conidia/mL, respectively.

**Figure 7:**
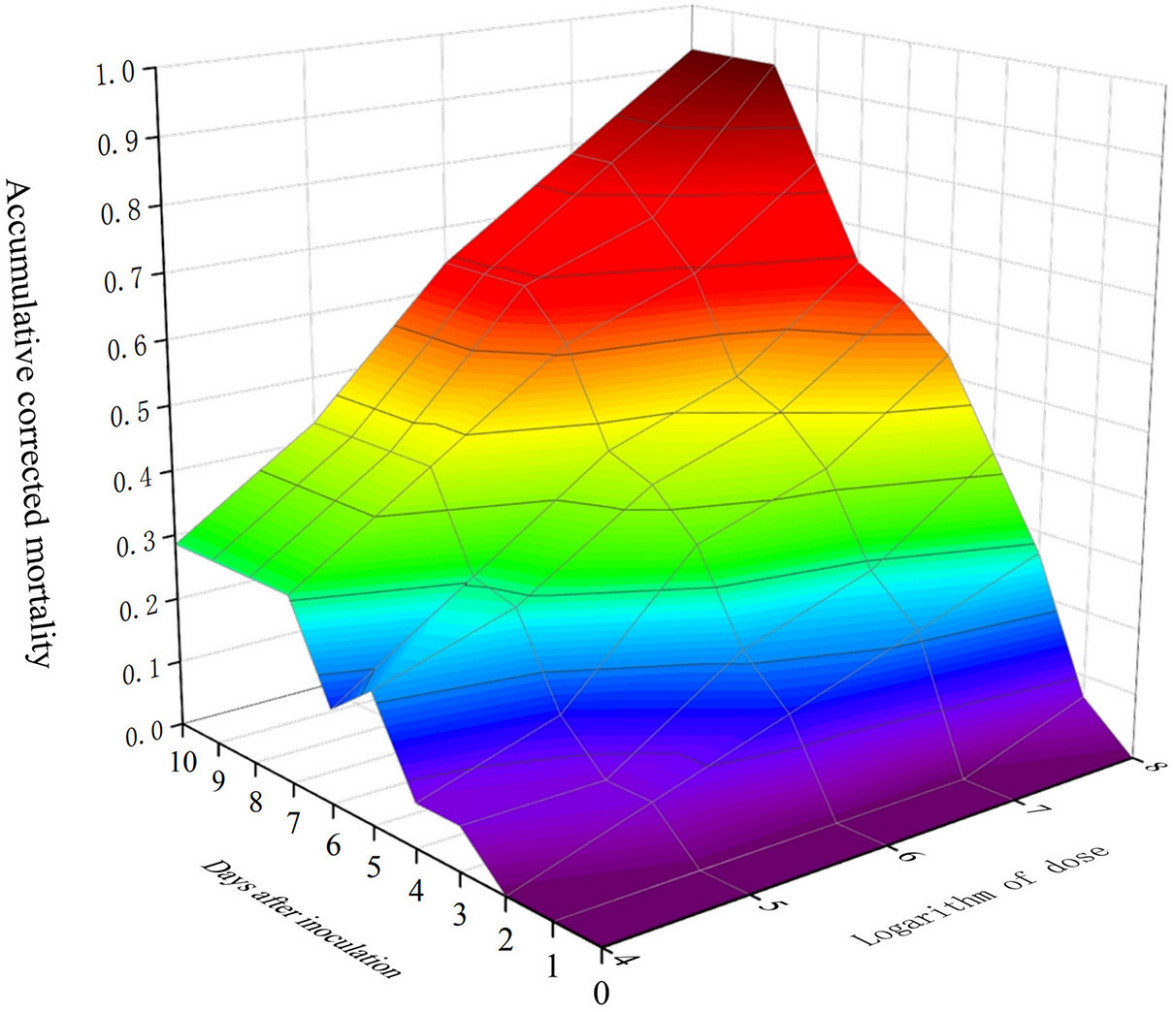
Three-dimensional plot mortality, time course, and fungal dose applied to a TDM model of *M*. *pingshaense* MaFZ-13 against *E. obliqua* in greenhouse experiments.

A time–dose–mortality (TDM) model was used to simulate and analyze the obtained data, with a data processing system (DPS) used to analyze the time–dose–mortality data of MaFZ-13 (Table 3). These data show that *M*. *pingshaense* MaFZ-13 (*chi* = 0.9943, d*f* = 8, *p* = 0.99828, *P* > 0.05) conforms to Hosmer–Lemeshow fit heterogeneity test, indicating that there is no significant difference between the predicted value and the practical value and accords with TDM model. Overall, the edge lines in the three-dimensional model directly reflected the maximum death possibility of the tested fungus (Figure 7), confirming the high virulence of *M*. *pingshaense* MaFZ-13 toward *E. obliqua*.

**Table 3:** TDM analysis of *M*. *pingshaense* MaFZ-13 against *E. obliqua*.

**Conditional mortality model**				**Cumulative mortality model**			
**Parameter**	**Value**	**SE**	***t-*** **test**	**Parameter**	**Value**	**Var (**τ**)**	**Cov (**τ, β**)**
β	5.8913	1.5965	3.6901	β	5.8913	0.651	0.651
γ1	−8.994	1.7408	5.1667	τ1	−8.994	0.7739	−0.5571
γ2	−6.6954	1.3598	4.9238	τ2	−6.5997	0.4727	−0.5391
γ3	−7.1381	1.376	5.1876	τ3	−6.14	0.4583	−0.5356
γ4	−6.6603	1.3543	4.9178	τ4	−5.6735	0.4492	−0.5334
γ5	−6.5036	1.3032	4.9905	τ5	−5.3116	0.4438	−0.5257
γ6	−69330	1.3819	5.0171	τ6	−5.1313	0.4324	−0.5255
γ7	−5.7901	1.2901	4.4882	τ7	−4.7142	0.4221	−0.5201
γ8	−6.407	1.3929	4.5997	τ8	−4.5453	0.4235	−0.521
γ9	−7.6208	1.6807	4.5334	τ9	−4.5002	0.4231	−0.5208
γ10	−7.6204	1.7366	4.388	τ10	−4.5435	0.4976	−0.6131

## 4. Discussion

The brewing of tea, *Camellia sinensis*, is one of the oldest and most popular beverages consumed worldwide. However, a large variety of different insects and pests can infest tea crops resulting in both direct damage and indirect damage via transmission of a range of microbial tea pathogens, which can lead to substantial economic losses (Chen and Chen, 1989). Significant efforts have been expended in identifying natural enemies of tea pests that include predators, parasitoids, bacteria, viruses, and fungi (Ye et al., 2014). Entomopathogenic microbes are effective against the tea green leafhopper, *Empoasca onukii* Matsuda, the tea mosquito bug, *Helopeltis theivora* Water, the red spider mite, *Oligonychus coffeae* Nietner, among many others (Idris et al., 2020). In particular, beneficial fungi, including various *Trichoderma* species, and the entomopathogenic fungi *Beauveria bassiana* and *Metarhizium anisopliae*, have shown potential for control of tea mites and termites that attack tea, i.e., the live-wood eating termite, *Microcerotermes beesoni* Snyder, and other insect pests (Kumhar et al., 2020; Deka et al., 2021). However, infestation by the tea geometrid, *Ectropis obliqua*, remains difficult to control, with current pest management strategies broadly applying large doses of chemical pesticides. This is increasingly becoming problematic due to adverse ecological and human health effects of these chemicals and the potential for the development of resistance to such chemical agents, as immune defense and detoxification mechanisms in *E. obliqua* to certain pesticides, e.g., pyrethroids, have been shown to be robust (Yin et al., 2021). In addition, the desire for organic tea limits options for these growers. Synthetic volatile attractants for male moths have been developed, although the extent to which these are effective in controlling insect pests in the field remains to be determined since these could likely be used as part of larger control strategies (Sun et al., 2016).

Entomopathogenic fungi represent naturally occurring biocontrol agents which are increasingly easier to manufacture, offer convenient formulation, and can even help promote plant health via associations with plant roots and other structures (Dara, 2019). Thus, the application of these fungi can offer safer and more cost-effective alternatives and/or can be used to help decrease reliance on more toxic chemical agents. Entomopathogenic fungi have successfully been used for controlling some tea pests, although more efficacious isolates would increase this potential (Yang et al., 2022). *Metarhizium* spp. are one of the most widely studied and applied insect fungal pathogens and have been used to suppress multiple pests of different orders in diverse environments (Fernández-Bravo et al., 2021; Sharma et al., 2023). Identification of fungal isolates from specific insect hosts offers a means for identifying strains particularly virulent toward the desired insect target and represents the initial work required for subsequent selection and application in pest biological control/integrated pest management (IPM) programs. Here, after screening and isolation from insect cadavers on tea plants and from tea-growing region soils, we report the identification of four new record species of *Metarhizium* in China. Our data show previously unreported species of *Metarhizium* that offer a reservoir for discovery and potentially more directed application. In support of this idea, from the cohort characterized, we have identified a highly virulent isolate of *M. pingshaense* toward *E. obliqua*. Previous records of *Metarhizium* species have described *M. anisopliae, M. brunneum, M. lepidiotae, M. majus, M. pinghaense*, and *M. robertsii* in China (Xu et al., 2016). Earlier reports with *M. pingshaense* strains, particular, have shown them capable of infecting and resulting in significant mortalities toward the rice leaf folder, *Cnaphalocrocis medinalis* (Kirubakaran et al., 2018), the subterranean termite, *Odontotermes obesus* (Francis, 2019), various mosquitoes species (Lovett et al., 2019), and the yellow peach moth, *Conogethes punctiferalis* (Senthil Kumar et al., 2021); however, our isolate appears to be an excellent pathogen of *E. obliqua*. Characterization of morphological parameters including colony characteristics, shape, and size of the conidiophores, phialides, and conidia is generally employed to determine the identification of (insect pathogenic) fungal species (Mongkolsamrit et al., 2020), and the morphological traits of the fungal isolates reported here accorded well with previous depictions for each respective species of *Metarhizium* reported (Chen et al., 2021; Fernández-Bravo et al., 2021; Wei et al., 2022). To further confirm strain identities, we used molecular phylogenetic analyses based on multi-gene sequences of the *TEF* and *RPB1*genes, which have been shown to afford higher level discrimination than *ITS, LSU*, and/or *tubulin* sequences for this genus, allowing for species assignment to our isolates (Kepler et al., 2014; Senthil Kumar et al., 2021; Paolo Barzanti et al., 2023).

In laboratory bioassays, the various fungi were found to cause 40–93% mortality toward *E. obliqua*, when tested at a concentration of 1 × 10^8^ conidia/mL indicating their different infective potentials and virulence against these insects as compared with a currently commercialized strain *M. anisopliae* CQMa421 which showed 79% mortality at the same concentration. Infected insects displayed signs of mycosis and copious sporulation 3–5 days post-mortem on host cadavers. High levels of sporulation produced on cadavers are a prominent feature of a successful biological control agent and can facilitate fungal spread under field conditions leading to epizootic infection and more effective control (Senthil Kumar et al., 2021). The median lethal concentration to kill 50% of infected insects (LC_50_) of the isolated highly virulent *M. pingshaense* MaFZ-13 strain against *E. obliqua* was calculated to be 9.6 × 10^4^ conidia/mL, and the mean lethal time to kill 50% of target hosts (LT_50_) values was between 4.3 and 5.3 days. For other insects, previous findings using (different isolates of) *M. pingshaense* had described LC_50_ values ranging from 2.3 × 10^5^ to 2.5 × 10^6^ conidia/mL and LT_50_ values in the range of 4.7–6.4 days against late instar larvae of the yellow peach moth, *Conogethes punctiferalis* (Senthil Kumar et al., 2021), and 3.9–4.4 days against fourth instar larvae of rice leaf roller, *C. medinalis* (Kirubakaran et al., 2018). Thus, our data show high relative efficacy vs. *E. obliqua*. Greenhouse experiments were used to validate the laboratory bioassays as these sometimes do not equate to good control in field experiments. These data show that *M. pingshaense* MaFZ-13 was effective under these conditions and that larger scale field experiments are warranted. In addition, future experiments combining this strain with low doses of chemical pesticides and/or other methods (trapping) could result in robust IPM management in tea crops. These data indicate that we have identified a potential biocontrol agent for *E. obliqua*, an important pest of many plants throughout the world. Additional testing of the potential synergistic effects of *M. pingshaense* on plant growth, as this fungus is associated with the plant rhizosphere (Fernández-Bravo et al., 2021), is also warranted.

## Data availability statement

The datasets presented in this study can be found in online repositories. The names of the repository/repositories and accession number(s) can be found in the article/Supplementary material.

## Author contributions

CW, YC, and JQ: conceptualization. CW and JZ: methodology and visualization. CW, JZ, and YL: software. HP, JL, and SL: validation. PL and MZ: formal analysis. CW, JZ, and YC: investigation. XH, SC, and JQ: resources. XG: data curation. JZ and JQ: writing—original draft preparation. NK and JQ: writing—reviewing and editing. JQ: supervision, project administration, and funding acquisition. All authors contributed to the manuscript revision, and read and approved the submitted version.
