# Supplementary material for: Isolation of a highly virulent Metarhizium strain targeting the tea pest, Ectropis obliqua
Source: Front Microbiol. 2023 May 15;14:1164511. doi: 10.3389/fmicb.2023.1164511 (PMC10226365; doi:10.3389/fmicb.2023.1164511)
Supplement: Supplementary file 1 [file Data_Sheet_1.PDF]

Supplementary Material

Isolation of a highly virulent *Metarhizium* strain targeting the tea pest, *Ectropis obliqua*

Jie Zhao, Yuxi Chen, Nemat O Keyhani, Cong Wang, Yichen Li, Huili Pu, Jincheng Li, Sen Liu, Pengyu Lai, Mengjia Zhu, Xueyou He, Shouping Cai, Xiayu Guan*, Junzhi Qiu*

Correspondence: Xiayu Guan, 47126940@qq.com; Junzhi Qiu, junzhiqiu@126.com

1 Supplementary Data

1.1 Supplementary Figures

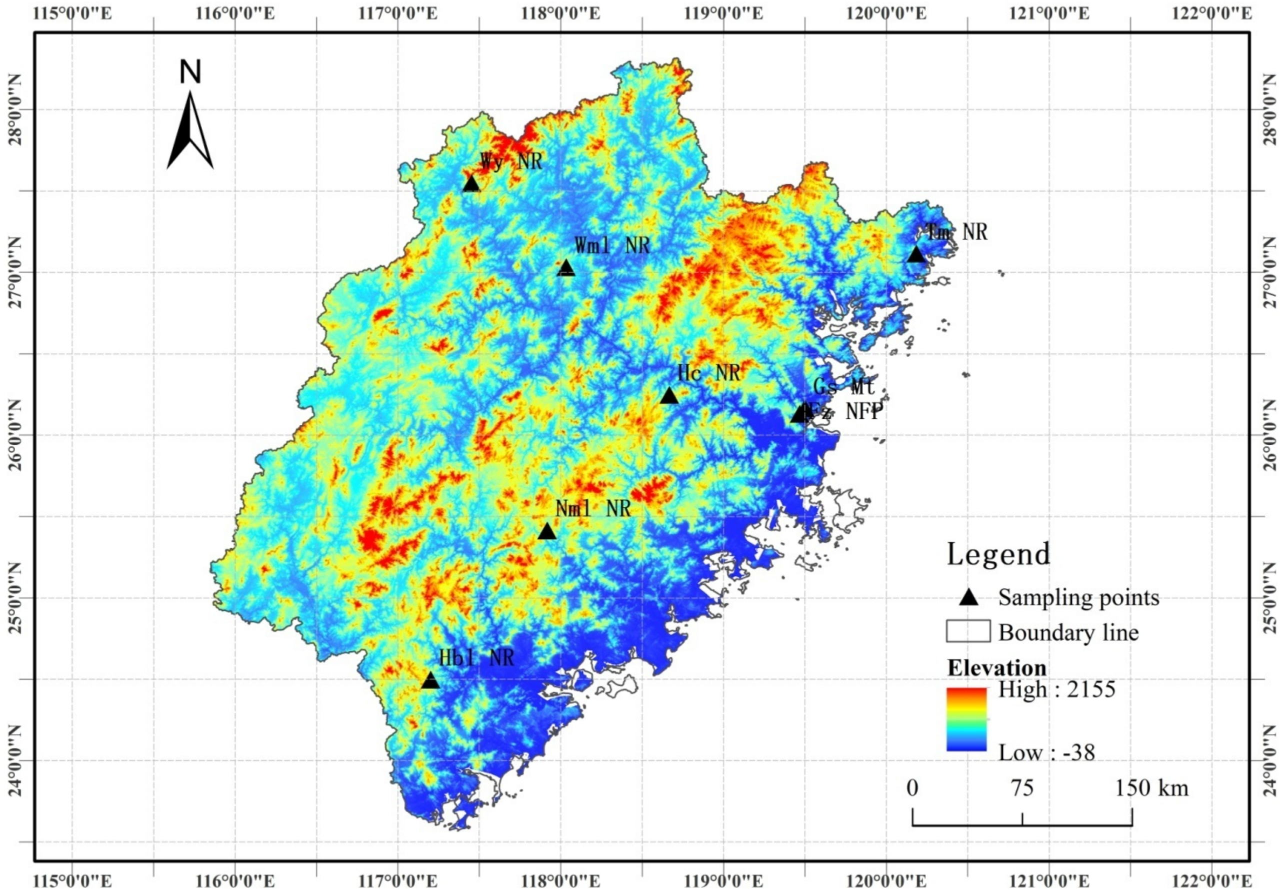

Supplementary Figure 1. Location map of sampling sites in Fujian province.

Note: WyNR, WmlNR, TmNR, HcNR, GsMt, FzNFP, NmlNR and HblNR stands for Wuyi Mountain Nature Reserve of Wuyi City, Wanmulin Nature Reserve of Jian'ou City, Taimu mountain Nature Reserve of Ningde City, Huangchu Forest Nature Reserve, Gushan Mountain of Fuzhou city,

Fuzhou National Forest Park, Niumulin Nature Reserve of Quanzhou City, and Hubo Liao Nature Reserve of Zhangzhou City, respectively.

1.2 Supplementary Tables

Supplementary Table 1. Summary data for the 11 strains of *Metarhizium* investigated in the present study

Strains	Host or origin	Species	TEF	RPB1
MaTGZ-01	Soil	*Metarhizium brunneum*	OL691548	OM022021
MaJO-01	Phasmatodea	*M. phasmatodeae*	OL691552	OL964990
MaQZ-02	*Acanthocinus*sp.	*M. robertsii*	OM022020	OL964985
MaTK-01	*Curculio chinensis*	*M. indigotica*	OL691550	OL964987
MaXJ-06	Curculionidae	*M. majus*	OL691555	OL964991
MaNATR-02	Soil	*M. clavatum*	OL691549	OL964986
MaYTTR-04-04	Soil	*M. anisopliae*	OL691547	OL964984
MaXJ-04-1	Curculionidae	*M. pingshaense*	OL943530	OL943531
MaFZ-11	Cerambycidae	*M. pemphigi*	OL691554	OL964989
MaQL-02	Coleoptera	*M. lepidiotae*	OL691551	OL964988
MaFZ-13	*Algedonia coclesalis*	*M. pingshaense*	OP903114	OQ269575

Supplementary Table 2. Typical characteristics of *Metarhizium* species on PDA in this study

Species	Phialides (μm)	Conidia (μm)	Colony description	References
Metarhizium anisopliae	Cylindrical,5-10.5×2-3	Cylindrical to ellipsoid, 5-9×2-3	White to olive	Kepler et al., 2014
M. brunneum	Cylindrical, 4.5-7×1.5-2	Oval,3.5-5×2-3	White to grayish green	Mongkolsamrit et al., 2020
M. clavatum	Cylindrical,4-9×1.5-3	Cylindrical,4-8×2.5-3	White to yellowish green	Mongkolsamrit et al., 2020
M. indigoticum	Cylindrical,6-9×2-3	Cylindrical,6.5-9.5×2-3	Dark brown	Kepler et al., 2014
M. lepidiotae	Cylindrical,6-10×2-3	Cylindrical,5-7×2-2.5	White to dark brown	Mongkolsamrit et al., 2020
M. majus	Cylindrical,6-10×2-3	Clavate,5-10×2.5-3	White to dark green	Mongkolsamrit et al., 2020
M. pemphigi	Cylindrical,5-7×1.5-2	Cylindrical,4-6×1.5-2	Light green	Kepler et al., 2014
M. phasmatodeae	Cylindrical,5-10×2-3	Cylindrical,5-9×2-3	Leaf green or olive	Mongkolsamrit et al., 2020
M. pingshaense	Cylindrical,5-17×2-3.5	Cylindrical to ellipsoid, 5-8.5×1.5-3.5	Olive	Mongkolsamrit et al., 2020
M. robertsii	Cylindrical,5-11×1.5-2	Clavate,5-9×2-3	White cream to green	Mongkolsamrit et al., 2020
